# Diversity of AMPA Receptor Ligands: Chemotypes, Binding Modes, Mechanisms of Action, and Therapeutic Effects

**DOI:** 10.3390/biom13010056

**Published:** 2022-12-27

**Authors:** Elena A. Golubeva, Mstislav I. Lavrov, Eugene V. Radchenko, Vladimir A. Palyulin

**Affiliations:** Department of Chemistry, Lomonosov Moscow State University, Leninskie Gory 1/3, 119991 Moscow, Russia

**Keywords:** glutamatergic system, AMPA receptor, modulators, antagonists, mechanism of action, molecular modeling

## Abstract

L-Glutamic acid is the main excitatory neurotransmitter in the central nervous system (CNS). Its associated receptors localized on neuronal and non-neuronal cells mediate rapid excitatory synaptic transmission in the CNS and regulate a wide range of processes in the brain, spinal cord, retina, and peripheral nervous system. In particular, the glutamate receptors selective to α-amino-3-hydroxy-5-methyl-4-isoxazolepropionic acid (AMPA) also play an important role in numerous neurological disorders and attract close attention as targets for the creation of new classes of drugs for the treatment or substantial correction of a number of serious neurodegenerative and neuropsychiatric diseases. For this reason, the search for various types of AMPA receptor ligands and studies of their properties are attracting considerable attention both in academic institutions and in pharmaceutical companies around the world. This review focuses mainly on the advances in this area published since 2017. Particular attention is paid to the structural diversity of new chemotypes of agonists, competitive AMPA receptor antagonists, positive and negative allosteric modulators, transmembrane AMPA regulatory protein (TARP) dependent allosteric modulators, ion channel blockers as well as their binding sites. This review also presents the studies of the mechanisms of action of AMPA receptor ligands that mediate their therapeutic effects.

## 1. Introduction

L-Glutamic acid (Glu, **1**) is one of the main excitatory neurotransmitters that also acts as a key neuromodulator regulating the functioning of synapses and neural pathways at various spatial and temporal scales [1,2,3]. The glutamatergic system plays a very important role in the operation of the mammalian central nervous system and the pathogenesis of many neurological and neurodegenerative diseases. The diversity of its functions is provided by a wide range of receptors belonging to two families: ionotropic glutamate receptors (iGluR), which are ligand-gated ion channels that conduct excitatory currents when exposed to glutamate, and metabotropic glutamate receptors (mGluR), which belong to the G protein-coupled receptors and control cellular processes via G protein-mediated signaling cascades. In turn, ionotropic glutamate receptors are divided into three subfamilies named after their selective agonists: α-amino-3-hydroxy-5-methyl-4-isoxazolepropionic acid **2** (AMPA), N-methyl-D-aspartic acid **3** (NMDA), and kainic acid **4** (KA) [2,3]. About 40 years have passed since the initial concept of excitatory amino acids and the role of glutamate as a neurotransmitter were established by Evans and Watkins [4,5], and AMPA was discovered as a selective agonist of a particular subpopulation of glutamate receptors [6]. The progress achieved in the field over this period, in terms of both understanding the processes and controlling them, is truly mind-boggling. However, great advances also reveal great complexity, and the potential for further development is far from exhausted.

While having a similar overall architecture, the ionotropic glutamate receptors differ significantly in their detailed structure, functions, and pharmacological characteristics. AMPA receptors (AMPARs) are present in the CNS in the largest quantities and feature the fastest signal transmission. They play a crucial role in providing synaptic plasticity, which is one of the mechanisms of learning and memory formation [7,8], and can also be targeted for the creation of new classes of drugs for the treatment or significant correction of a number of serious neurodegenerative and neuropsychiatric diseases, such as epilepsy, Alzheimer’s disease, Parkinson’s disease, multiple sclerosis, mild cognitive disorders, age-related cognitive and memory impairments, autism, depression, drug addiction, etc. [2,9]. Taking this into account, the search for various types of AMPA receptor ligands and the studies of their properties attract considerable attention from both academic institutions and pharmaceutical companies around the world. This review focuses mainly on the advances in this area published since 2017; the results of the earlier studies are sometimes mentioned if logically desirable. They are systematically considered, for example, in reviews [7,10,11,12,13].


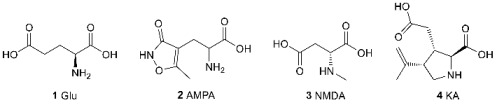


## 2. AMPA Receptor Architecture and Mechanism of Operation

**Figure 1:**
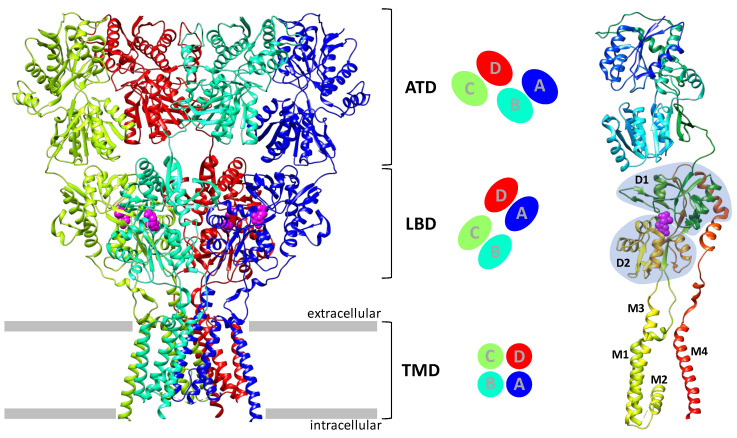
AMPA receptor architecture: overall receptor structure (PDB: 5VHZ) and its location in the membrane (left; individual subunits are colored blue, mint, spring green, and red); subunit contact topology and symmetry of the amino-terminal, ligand-binding, and transmembrane domains (center); structure of an individual subunit (right; backbone chain colored using “rainbow” color scheme). The location of the binding site for agonists and competitive antagonists is shown in purple; the D1 and D2 lobes of the ligand-binding domain as well as the transmembrane segments M1–M4 are marked in the subunit structure.

Similar to other ionotropic glutamate receptors, the AMPA receptor is a tetrameric protein consisting of four subunits that form a cation channel (Figure 1). Each subunit consists of the extracellular amino-terminal domain (ATD), the ligand-binding domain (LBD) containing an agonist binding site (the term “agonist binding domain”, ABD, although more precise, is not yet commonly used), the transmembrane domain (TMD) that forms the ion channel pore, and the intracellular C-terminal domain (CTD) [3,14,15]. The transmembrane domains form a symmetrical tetramer, while the ligand-binding and amino-terminal domains have the structure of a dimer of dimers, with different topologies of contacts between their subunits.

There are four types of AMPA receptor subunits (GluA1–GluA4), which can combine to form homo- or heterotetramers. Diheterotetrameric receptors of the composition GluA1–GluA2 and GluA2–GluA3 are most common in the mammalian brain [16]. All subunit types differ slightly in channel operation kinetics, selectivity, and trafficking properties, resulting in significant structural and functional diversity within the receptor population. This diversity is further enhanced by numerous options for alternative splicing, post-transcriptional and post-translational modification. Alternative splicing in the ligand-binding domain gives rise to the so-called “flip” and “flop” isoforms that differ in several amino acid substitutions at the LBD subunit interface, differentially affecting receptor kinetics and sensitivity to modulators [10,17]. Of particular importance is the post-transcriptional RNA editing that leads to the substitution of glutamine by arginine in the ion channel pore of the GluA2 subunit (at the end of the transmembrane loop M2) which affects channel permeability to calcium ions and its block by endogenous polyamines [18,19,20]. Most AMPA receptors contain the GluA2 subunit and are permeable only to Na^+^, but not to Ca^2+^. However, a small but important part of the receptor population does not contain this subunit (or it is not modified), making such receptors permeable to calcium and contributing to the development of excitotoxicity. Resembling the NMDA receptors, which they indirectly interact with, such receptors may play a crucial role in synaptic plasticity in several neurodegenerative diseases, including Alzheimer’s disease and schizophrenia [18,20,21,22]. Post-translational modifications, especially phosphorylation, ubiquitination [23], and palmitoylation [24], also significantly affect the regulation of receptor trafficking and synaptic plasticity.

Due to the great variety of subunit stoichiometry in native AMPA receptors, most studies are carried out either on model receptors of a certain composition (usually GluA2 tetramer) or on an average population of receptors [3]. However, the results of studies using cryo-electron microscopy and the antibody fragments specific to certain subunits have recently been published, which opened the way to the elucidation of stoichiometry and the arrangement of subunits in native AMPA receptors [16]. These data made it possible to identify 10 main types of subunit stoichiometry and discovered that the GluA2 subunit usually occupies the B and D positions, while the GluA1 and GluA3 subunits occupy the A and C positions. Additionally, the previously undescribed triheterotetrameric receptors of the A1A2A3A2 composition, which make up a significant part of the receptor population, were found.

**Figure 2:**
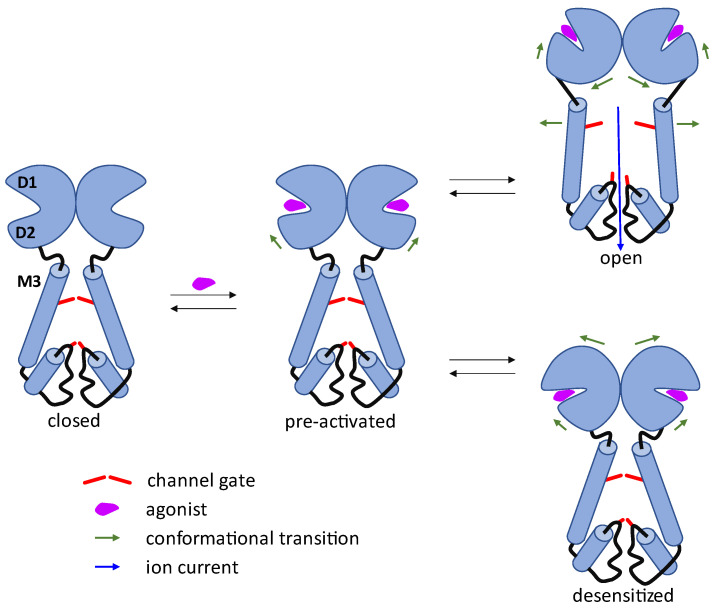
Simplified scheme of the AMPA receptor mechanism of operation (the ion channel opening and closing).

Over the past few years, significant progress in understanding the mechanism of operation of the AMPA receptor and, in particular, the opening and closing of the ion channel, has been made. These results are driven primarily by the development of X-ray crystallography and cryo-electron microscopy methods, which made it possible to determine a number of complete receptor structures simulating their various states and to analyze their conformational differences [15,25]. A simplified scheme of the processes occurring upon binding of an agonist to the receptor is shown in Figure 2. A key role in the receptor gating is played by the ligand-binding domain that is shaped as a clamshell, inside of which (between the D1 and D2 lobes) the agonist binding site is located. In an unbound receptor in the closed (resting) state, the M3 transmembrane helices are drawn together, closing the channel pore. When an agonist binds, the clamshell partially closes due to the shift of the D2 lobe towards the D1 lobe, and the receptor is transferred to the pre-activated state, in which the channel is still completely closed. As the D2 and D1 lobes are further drawn together during the clamshell closing, while maintaining the interaction of the “back” parts of the clamshells in the dimer, this causes substantial separation of the D2 lobes leading to the strain of the linkers between the LBD and TMD, and, as a result, the separation of the M3 transmembrane helices from each other and the opening of the channel gate. When the agonist is unbound, the receptor is transferred from the pre-activated state to the closed state.

An important feature of the AMPA receptor is its tendency to desensitization, i.e., the closing of the channel (or reducing its permeability) while maintaining agonist binding. This process limits the duration of activation and the magnitude of the total ion current, and can also affect the intracellular trafficking of the receptor [3,15]. Desensitization is explained by the fact that in the closed conformation of the clamshells, it is thermodynamically more favorable to rotate their back parts relative to each other, which leads to the separation of the D1 lobes from each other, the drawing of the D2 lobes together, the weakening of the linkers tension, and the stabilization of the closed state of the transmembrane domain [15,25]. The transition of the receptor from the open state to the desensitized state cannot occur directly but can only go through the pre-activated state. Recently it was found that in the GluA2 tetramers with the substitution of glutamine by arginine (GluA2(R)), the desensitization proceeds more slowly and a significant transmembrane current is observed in the desensitized state. A hypothetical explanation for this phenomenon involves the interaction of the arginine residues in the ion channel pore that prevents the channel from closing during desensitization [26]. The study of conformations and kinetics of the AMPA receptor desensitization indicates the existence of a hierarchy of desensitized states [27]. The more substantial rearrangements of the quaternary structure likely correspond to stable desensitized states that are adopted relatively slowly and may occur in brain injury or outside of the fast excitatory synapses. On the other hand, slightly disrupted structures are rapidly attained by AMPA receptors within milliseconds, making them relevant for desensitization in the brain.

In recent years, the important role of the so-called AMPA receptor auxiliary subunits (ARASs, sometimes called auxiliary proteins) has been finally recognized [14,25,28,29,30]. These proteins form receptor signaling complexes with AMPARs and can modulate their trafficking, opening, closing, desensitization, pharmacological properties, and permeability, providing fine-tuning of the AMPA receptor functions in space and time.

It has been shown that the presence of certain auxiliary subunits (or their absence in the study of recombinant receptors) can significantly affect the observed activity of various classes of receptor ligands [25,31,32], and the ligands selective to the complexes of the AMPA receptor with certain proteins provide the opportunity of targeted control of the pharmacological effects [33,34].

Currently, the AMPA receptor auxiliary subunits include a wide range of transmembrane proteins structurally unrelated to the receptor that belong to several groups [25,28]:

- Transmembrane AMPA receptor regulatory proteins (TARP), including the first discovered stargazine (γ2), γ3, γ4, γ5, γ7, and γ8. Their structure includes four transmembrane helices and a number of extracellular loops containing β-sheet fragments and disordered regions. The formation of complexes with TARP potentiates the AMPA receptor currents;
- Germ cell-specific gene 1-like protein (GSG1L) is similar to TARP in overall structure (allowing one to assign these two groups to the claudin family) but has an “inhibitory” effect on the receptor;
- Cornichon homolog (CNIH) proteins contain four transmembrane helices, with the spatial structure of their transmembrane domain being very similar to that of TARP (despite the different helix arrangement), but do not contain an extracellular domain;
- Cysteine-knot AMPA receptor modulating proteins (Shisa/CKAMP family) include one transmembrane domain, an extracellular cysteine-knot motif, and a long intracellular C-terminal tail;
- The SynDIG1 and SynDIG4 proteins (Dispanin C family) contain a single transmembrane domain and a long extracellular C-terminal tail with a membrane-associated domain.

A broad phylogenetic analysis demonstrates that these four unrelated families have rather ancient origins; however, the majority of ARASs has emerged during vertebrate evolution by independent convergent processes of neo/subfunctionalization of unrelated proteins that resulted in the presence of multiple ARASs [35]. It is conjectured that the increase in the ARAS repertoire, and the concomitant increase in the AMPAR functionalities, contributed to the growing complexity of vertebrate brains and cognitive functions.

**Figure 3:**
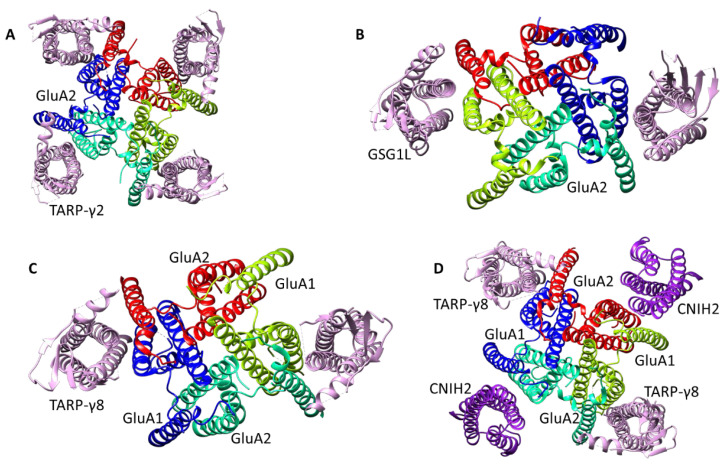
Representative examples of experimentally determined structures of AMPA receptor complexes with auxiliary subunits. (**A**) GluA2 + 4 TARP-γ2 (PDB: 5VOT), (**B**) GluA2 + 2 GSG1L (PDB: 5WEM), (**C**) GluA1/2 + 2 TARP-γ8 (PDB: 6QKZ), (**D**) GluA1/2 + 2 TARP-γ8 + 2 CNIH2 (PDB: 7OCA).

The TARP, GSG1L, and CNIH proteins are the best investigated and appear to be more common in different regions of the brain. According to the cryo-electron microscopy data, they form complexes with the AMPA receptor containing up to four auxiliary subunit molecules, with each of them associated with the transmembrane domain of an individual receptor subunit (Figure 3) [25,28]. It is still not entirely clear which of the possible stoichiometries of the complexes are realized under physiological conditions and/or are sufficient for the manifestation of the observed effects. 

TARP and GSG1L extracellular loops can interact with the ligand-binding domain of the receptor [36,37]. It is believed that TARP can prevent a significant structural rearrangement of the ligand-binding domains in the desensitized receptor and thereby facilitate the restoration of the dimer–dimer configuration (or even promote resensitization), while GSG1L stabilizes the desensitized receptor in conformations incompatible with channel opening due to its interaction with extracellular domains [28,37]. The available data on the additional TARP interaction with the amino-terminal domain [38] have not yet received a structural explanation.

In addition to the auxiliary subunits, the AMPA receptors are known to dynamically interact with many other intracellular, presynaptic, synaptic, and postsynaptic proteins [39,40,41,42]. In fact, the synaptic environment is quite dense and tight, and the receptors never function as isolated stand-alone units. Moreover, the glutamatergic cycle is very fastpaced, imposing a millisecond timescale on receptor activation, deactivation, and desensitization. It is believed that such rapid switching is enabled by the high conformational flexibility of the ligand-binding domains that are connected to other domains by flexible linkers [39]. The complex AMPAR interaction networks are still being gradually unraveled and only partially understood. Nevertheless, it is now clear that they critically influence receptor operation, regulation, trafficking, synaptic plasticity, and long-term potentiation, and may be disrupted in certain diseases [39,40,41,42].

## 3. Binding Sites and Ligand Types of the AMPA Receptor

**Figure 4:**
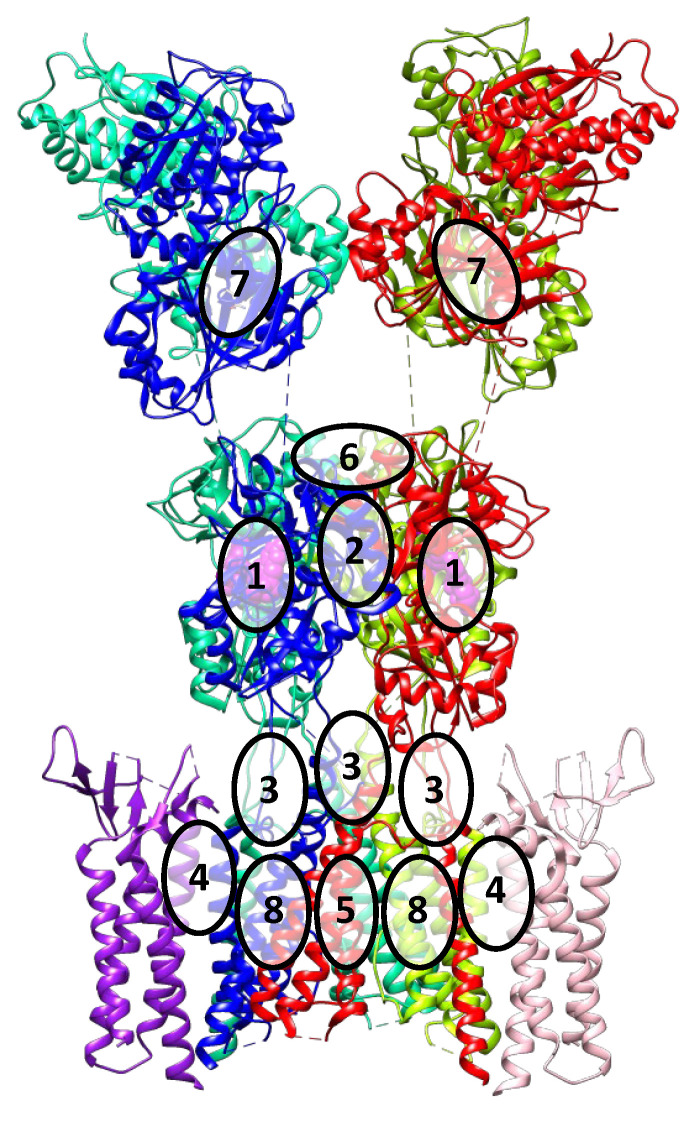
Various binding sites in the AMPA receptor. Similar to Figure 1, the receptor subunits (PDB: 6QKZ) are shown in blue, mint, spring green, and red, while the competitive antagonist is shown in magenta. The representative auxiliary subunits (TARP γ8) are shown in pink and purple. For the numbering of the sites, see the text.

Compared to the NMDA receptor, fewer ligand binding sites are known for the AMPA receptor [2,3]. They are briefly described below, and their approximate locations are shown in Figure 4.

(1). The orthosteric binding site for agonists and competitive antagonists is located inside the clamshell of the ligand-binding domain;
(2). The positive allosteric modulator binding site is located at the interface between the subunits of the dimeric ligand-binding domain;
(3). The binding site for negative allosteric modulators (non-competitive antagonists, non-competitive inhibitors) is located in the linker region between the ligand-binding and transmembrane domains;
(4). The binding site for TARP-dependent allosteric modulators is located at the interface between the interacting transmembrane segments of TARP and the receptor;
(5). Ion channel blocker binding sites are located in the pore of the ion channel (mainly for the Ca^2+^-permeable forms of the receptor);
(6). Con-ikot-ikot, a protein toxin from the *Conus striatus* cone snail, acts as an AMPAR positive allosteric modulator by binding on top of the LBD dimer-of-dimers (in the free space between LBDs and ATDs). This effectively immobilizes the LBD layer of the receptor and prevents desensitization, leading to prolonged receptor activation (although mostly to only partially open states), over-excitation, and toxicity [3,43]. It is not yet clear if this binding site could be exploited by the small molecule ligands.
(7). Based on molecular modeling and structural data, another potential binding site is predicted to be located at the interface between the lower lobes of the subunits of the dimeric amino-terminal domain [44]. Presumably, this site is present (or druggable) only in the GluA3 receptors, while the ligands interacting with it are not known yet. Its position at the dimer interface is similar to that of the binding sites in related NMDA receptors, as well as in metabotropic glutamate receptors. Taking into account the specificity of the amino acid sequence of the AMPA receptor amino-terminal domain, these data make it a promising potential target for the development of new modulators with high selectivity.
(8). Based on the analysis of the structure of the AMPA receptor complex with *trans*-4-butylcyclohexane carboxylic acid (4-BCCA), new binding sites were found in the transmembrane domain. Driven by the structural data as well as site-directed mutagenesis and molecular modeling, three possible mechanisms of action of 4-BCCA were proposed that involve either direct blocking of the ion channel (interfering with the flow of permeant ions), or influencing the dynamics of the M3 helices, or destabilizing the protein surface through competition with the surrounding membrane lipids [45]. Presumably, other related compounds which are inhibitors of the AMPA receptor also bind at these sites and can serve as promising potential antiepileptic drugs. Moreover, these sites are located in close proximity to those for the negative allosteric modulators, which opens the possibility of interaction of the respective structural fragments and could explain the synergistic effect observed with the simultaneous administration of perampanel and decanoic acid [46].

There are also reports on the modulation of AMPA receptors by Zn^2+^ ions [47,48] and nitric oxide (NO) [49].

It should be noted that the receptor can simultaneously and specifically bind several ligands at different sites, for example, competitive antagonists and negative allosteric modulators [50] or various auxiliary proteins [28]. In some cases, cooperative effects are observed upon ligand binding, which is amenable to kinetic modeling [51]. The receptors also can open to multiple conductance states that vary between the specific receptor subpopulations (among other things, they depend on the subunit composition, protein modifications, and TARP partners) [52]. The postsynaptic currents can be represented by a data-driven conductance model [53]. Finally, antagonists selective to certain subunits can cause overall potentiation of the receptor by reducing the agonist binding and, as a result, attenuating desensitization [54].

The main results of studies of these sites and their ligands obtained in recent years will be considered below.

### 3.1. The Orthosteric Binding Site for Agonists and Competitive Antagonists

As explained above, the orthosteric site is located inside the “clamshell” of the ligand-binding domain and provides the binding of full and partial agonists as well as competitive antagonists of the receptor. Although extensive research on its ligands has already been performed in previous years, it still attracts attention as a target for the development of potential drugs and/or pharmacological tools.

It should be noted that AMPA receptor agonists can enhance desensitization and often cause a number of side effects, including seizures and neurotoxicity [55]. In this regard, the development of competitive receptor antagonists is more promising for clinical use.

The development of the quinoxaline-2,3-dione scaffold, a classic one for competitive AMPA receptor antagonists, has made it possible to obtain a large series of analogs with different selectivity profiles for ionotropic glutamate receptors, including a selective AMPA receptor antagonist **5** [56]. A detailed analysis of the binding of quinoxaline-2,3-diones showed that these compounds (for example, dinitro-derivative DNQX **6**) are easily deprotonated at pH close to physiological and interact with the receptor specifically in the anionic form [57]. By modifying this scaffold, reversible photoswitchable antagonists such as ShuBQX-3 (**7**) were developed (only the *trans*-form has antagonistic activity; it is formed upon irradiation by light with a wavelength of 600 nm and turns into an inactive *cis*-form at 400–500 nm, making it possible to accurately and non-invasively control the receptor operation in space and time) [58]. Based on the combination of quinoxaline-2,3-dione and kynurenic acid scaffolds, a series of hybrid quinazolinediones was constructed, including the potential antiepileptic drug selurampanel (**8**) [59].

Recently, new chemotypes of AMPA receptor agonists and competitive antagonists were being actively developed. For example, conformationally restricted bicyclic analogs of glutamic acid CIP-AS (**9**) and LM-12b (**10**) [60] act as partial or full agonists of the AMPA and kainate receptors, with a strong preference for the kainate GluK3 subunits. Their selectivity profiles and binding modes were analyzed using X-ray data. 

Based on the scaffold of aryl- and hetaryl-substituted phenylalanines, a series of competitive AMPA receptor antagonists such as compound **11** was obtained, their possible binding modes were analyzed using molecular docking, and their anticonvulsant and antioxidant properties were confirmed [61,62,63].

By modifying the structure of the natural compound (S)-willardiine **12**, which is a partial agonist of the AMPA receptor, a series of bicyclic derivatives of pyrimidinedione **13** was created [64,65]. It has been demonstrated that the replacement of the heterocyclic fragment leads to a significant (up to 500-fold) change in affinity. Molecular modeling and analysis of structural data obtained by X-ray crystallography have shown that this effect is caused by a significant difference in interactions with the protein and, especially, the binding site water molecules.

In the development of a series of glutamate and aspartate analogs **14–16**, the 4-hydroxy-1,2,3-triazole fragment was used as a bioisostere for the distal carboxylic group [66]. Compounds **14a–c** and **15a** were shown to be selective AMPA receptor agonists; moreover, compound **14b** exhibited selectivity to certain types of AMPA receptor subunits.


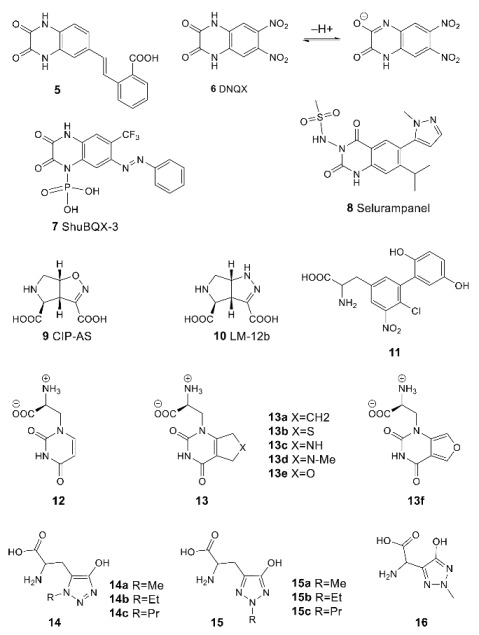


### 3.2. The Positive Allosteric Modulator (PAM) Binding Site

Positive allosteric modulators (PAMs) of the AMPA receptor (ampakines) bind at an allosteric site located at the interface between the subunits of the dimeric ligand-binding domain [7]. It is believed that their potentiating effect on the receptor is caused by the stabilization of its open form and/or the decrease in desensitization. Such modulators attract much attention due to their ability to improve learning and memory formation processes as well as their neuroprotective effect, which make them promising candidates for the development of drugs for the treatment of cognitive disorders (including the early stages of the Alzheimer’s disease), depression, and a number of other CNS pathologies [7,8,13] as well as the drug-induced respiratory depression [67]. The key advantage of the positive allosteric modulators of AMPA receptors is that, unlike agonists, they only potentiate the receptor in the presence of an endogenous ligand and therefore lead to fewer side effects [55,68]. 

However, in spite of the encouraging preclinical results, the majority of PAMs to date have not progressed beyond early clinical development [69]. Some of the problems in this field involve the insufficiently relevant in vitro and in vivo models of neurological and neuropsychiatric disorders, the pharmacodynamics and pharmacokinetics issues, and limited understanding of the processes underlying the activity and specificity of modulator action. For instance, in spite of significant progress in the development of the cell models [70] and animal models [71,72,73,74] for neurodegenerative diseases such as Alzheimer’s disease, amyotrophic lateral sclerosis, Parkinson’s disease, Huntington’s disease, as well as schizophrenia, no one of them is yet able to fully capture all the critical aspects and factors involved in these complex pathologies—especially as those factors are not yet fully understood. Nevertheless, the AMPA receptors remain an attractive target that has several unique advantages over other drug targets, and it is believed that the problems can be solved. Thus, identifying novel PAM chemotypes has become a priority for many pharmaceutical companies, and AMPAR PAMs offer many promising treatment avenues for neurological and neuropsychiatric disorders [69].

**Figure 5:**
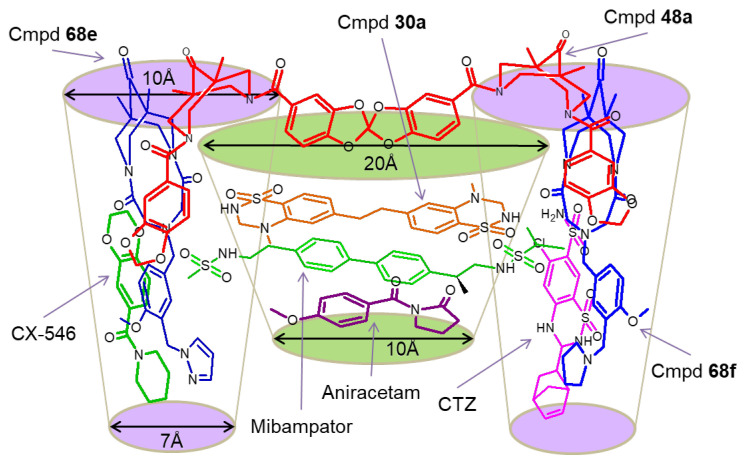
The overall architecture of the AMPA receptor positive allosteric modulator binding site and the location of some key modulators according to the X-ray diffraction analysis, molecular docking, and molecular dynamics. The central pocket is marked in green; the symmetrical side pockets are marked in purple.

From the structural perspective, reflecting the overall symmetry of the dimeric AMPAR ligand-binding domain, the positive allosteric modulator binding site has a symmetrical structure, including the central and two side pockets, and molecules of quite different sizes and chemical structures—from small nootropic compounds (for example, aniracetam **17**) to rather large and complex ones—can bind in its various regions (Figure 5) [7]. Perhaps this is one of the reasons that hinder the development of general principles of PAM molecular design, despite the structure–activity relationships found in individual series of compounds, the vast available experimental data on the structure of the modulator–receptor complexes (recently analyzed in deep detail [75]), and the success of molecular modeling using pharmacophore analysis, 2D and 3D QSAR studies, molecular docking, and molecular dynamics [2,76,77,78,79].

The classical chemotypes of the positive allosteric modulators of AMPA receptors are benzamides, benzothiadiazines, biarylalkylsulfonamides, and trifluoromethylpyrazoles.

Among the first AMPAR positive allosteric modulators were benzamides based on aniracetam **17**. The most promising molecules of this chemotype were CX516 (**18**), CX614 (**21**), CX691 (**22**), and CX717 (**23**) [80,81] (the only difference between the two latter structures is the replacement of the piperidine fragment in CX691 by morpholine). Later, the more distant racetam analogs were obtained such as S47445 (CX1632, tulrampator) **24** [82] and **25** [83]. 

**Figure 6:**
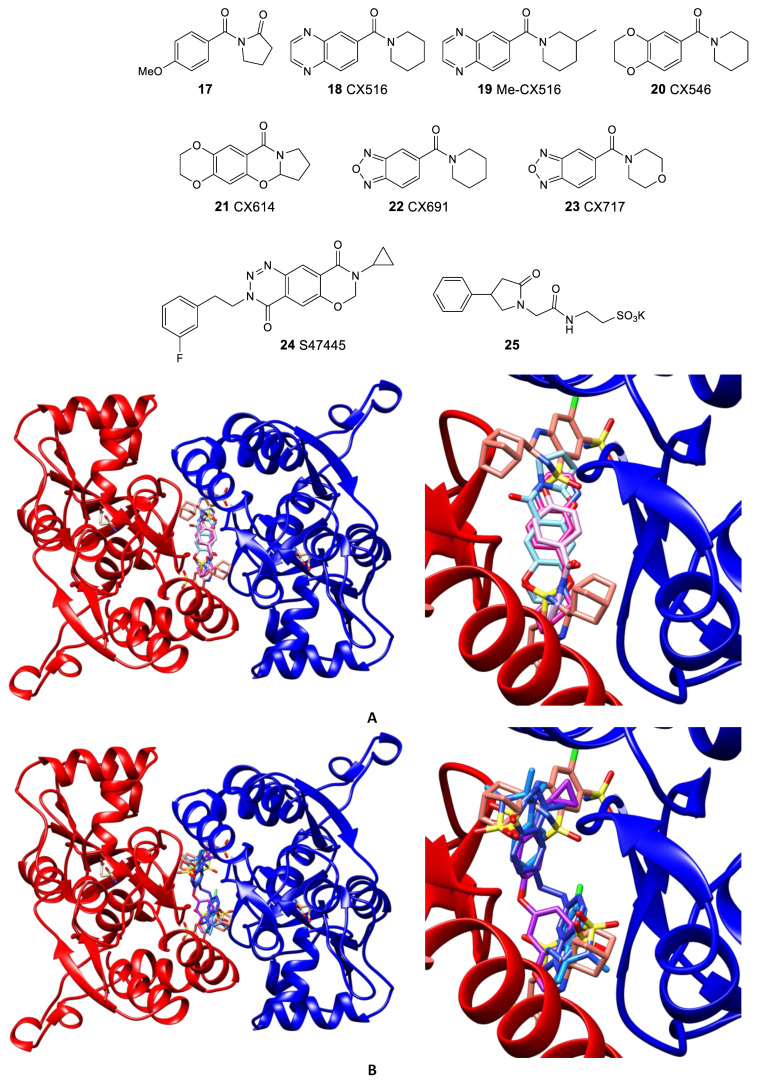
Comparison of the binding modes of AMPA receptor positive allosteric modulators in the dimeric LBD of GluA2 (unless stated otherwise). (**A**) aniracetam (**17**, pink, GluA3, PDB: 3LSW), CX516 (**18**, fuchsia, PDB: 4IY5), CX614 (**21**, cyan, PDB: 2AL4), and cyclothiazide (**26**, salmon, PDB: 3TKD). (**B**) cyclothiazide (**26**, salmon, PDB: 3TKD), BPAM-321 (**27**, dodger blue, PDB: 5BUU), BPAM-538 (**29**, purple, PDB: 5OEW), and TDPAM01 (**30a**, cobalt blue, PDB: 6FAZ). Left—general view, right—detailed binding mode. For clarity, only one symmetrically equivalent orientation is shown for each ligand. The aligned receptor subunits are shown in blue and red, and the agonist (glutamate) molecules bound in the orthosteric binding sites are shown in beige.

The analysis of the AMPAR LBD complexes with aniracetam, CX516 (**18**), Me-CX516 (**19**), and CX614 (**21**) [80] has shown that all these molecules bind in the central subpocket in a very similar way, with a key role played by the hydrogen bonds with the network of water molecules that mediate the interaction of a ligand with amino acid residues in the binding site (Figure 6A).

To date, the most significant therapeutic effects of CX516 have been shown in the animal models of intellectual disability (at 5 mg/kg dose for 5 days) [84] and hyperactivity commonly observed in schizophrenia and autism spectrum disorder (at 10–40 mg/kg) [85]. In recent studies, CX546 has shown reasonably good results in the autism models and was found to effectively mediate neurogenesis and dendritogenesis [86,87]. However, its low oral bioavailability limits the clinical development of this agent. In its turn, during preclinical studies, CX614 has shown itself as a promising brain-derived neurotrophic factor (BDNF) inductor [88] and enhanced the effects of antidepressants such as imipramine and reboxetine.

CX691 was studied for its procognitive effects in an animal model of Alzheimer’s disease, and increased hippocampal BDNF expression and improved spatial learning and memory were found [89]. For CX717, it has been established that it acts as a PAM and has a strong antidepressant effect [90]; in addition, CX717 is considered as a potential agent for the treatment of attention deficit hyperactivity disorder (ADHD).

In recent preclinical studies, it was found that S47445 (CX1632, tulrampator) significantly enhances synaptic plasticity and increases neurotrophin levels both in the hippocampus and the prefrontal cortex of aged mice [82,91]. Interestingly, the antidepressant and anxiolytic effects were also detected for this ligand in three animal models. These behavioral effects were accompanied by increased levels of hippocampal neurogenesis and BDNF [92,93]. S47445 has also shown procognitive effects in animal models [94,95,96]. Based on these results, Phase I clinical studies have been started for S47445 as a potential agent for the treatment of Alzheimer’s disease and dementia-associated depression (NCT02626572, NCT02805439). However, the results of the first double-blind placebo-controlled clinical study in patients with mild to moderate Alzheimer’s disease and depressive symptoms suggested that S47445, although well tolerated, did not show significant improvement in cognitive functions [97].

For compound **25**, extensive in vitro and in vivo preclinical studies have been conducted, confirming that it acts as a neuroprotective agent and can significantly reduce neurological deficits and restore cognitive functions after ischemic brain injury [83].

Although the derivatives and analogs of benzothiadiazine dioxide, starting with cyclothiazide (CTZ **26**), represent one of the most thoroughly researched classes of the AMPAR positive allosteric modulators, their studies continue to attract considerable interest. During the search for new chemotypes of modulators and the investigation of their structure–activity relationships, a number of highly potent structures were found, for example, **27** [98], **28a** and its unsaturated metabolite with comparable activity **28b** [99]. In some cases, the “unexpected” structure–activity relationships discovered during the study could be explained using thermodynamic analysis, experimental data on the binding modes, and molecular modeling [100,101]. Unlike most of the studied benzothiadiazine dioxides, which admit independent binding of two modulator molecules in different regions of the binding site at the LBD dimer interface, the larger molecules of the phenoxy derivative **29** [102] and the specially designed dimeric compounds **30** [76] occupy several regions of the site by binding of one molecule (Figure 6B) and exhibit activity in the nanomolar range.

Recently, an attempt was made to develop new positive allosteric modulators by switching from the benzothiadiazine dioxide scaffold to the thiochromane dioxide scaffold using a classical bioisosteric replacement [103]. However, the resulting series of compounds **31** exhibits lower activity toward the AMPA receptor compared to the original benzothiadiazine dioxides.


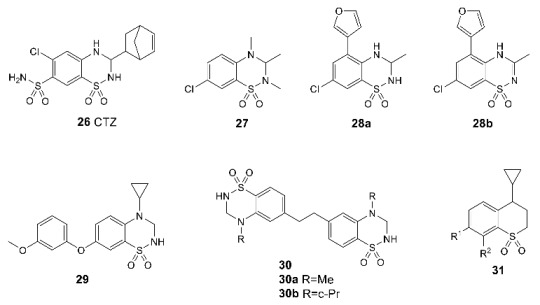


The first biarylalkylsulfonamide modulator, compound **32**, was found by high-throughput screening. Based on its structure, a number of analogs were created and detailed SAR studies were carried out. One of the most promising compounds exhibiting high activity and selectivity was LY-404187 (**33**, racemate) and its optically pure (R)-isomer LY-451646. In the earlier publications, its therapeutic effect was explained primarily by its antidepressant action clearly observed in the forced swim and the tail suspension tests [104,105]. 

**Figure 7:**
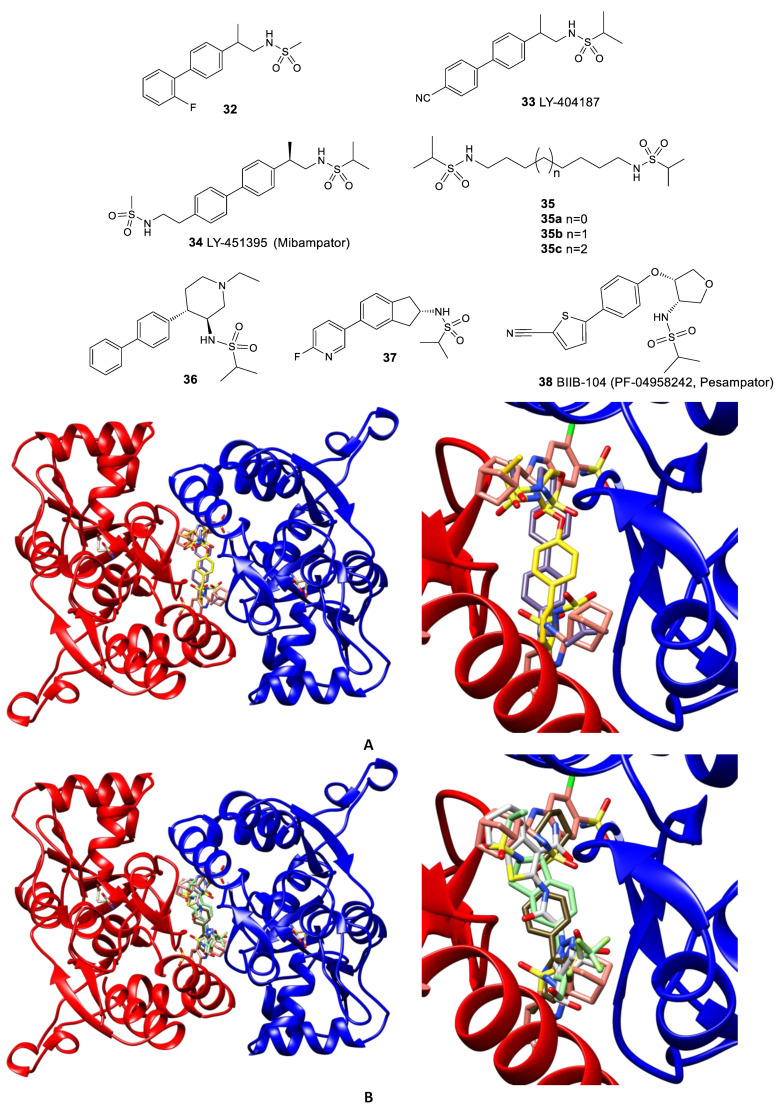
Comparison of the binding modes of AMPA receptor positive allosteric modulators in the dimeric LBD of GluA2. (**A**) cyclothiazide (**26**, salmon, PDB: 3TKD), LY-451395 (**34**, pale purple, PDB: 5YBG), and BIIB-104 (**38**, yellow, PDB: 4X48). (**B**) cyclothiazide (**26**, salmon, PDB: 3TKD), JAMI1001A (**41**, light grey, PDB: 4FAT), compound **43a** (pale green, PDB: 3PMW), and TAK-653 (**47**, dark olive, PDB: 7F3O). Left—general view, right—detailed binding mode. For clarity, only one symmetrically equivalent orientation is shown for each ligand. The aligned receptor subunits are shown in blue and red, and the agonist (glutamate) molecules bound in the orthosteric binding sites are shown in beige.

Further studies were aimed at developing dimeric structures (LY-451395 **34** and compounds **35**) wherein, according to the X-ray diffraction data, sulfonamide groups bind to the symmetrical side pockets and the biaryl fragment occupies the central pocket [81] (Figure 7A). Interestingly, the replacement of the biaryl fragment by alkyl chains of different lengths does not lead to a decrease in activity, which makes it possible to vary the structure of the linker over a wide range. Compound LY-451395 (mibampator **34**) was under phase II clinical trials for the treatment of Alzheimer’s disease, but the trials were terminated [106]. An alternative approach to the optimization of biarylalkylsulfonamides involves the creation of their conformationally restricted analogs (for example, compounds **36**, **37**, and BIIB-104 **38**). Among them, the compounds with an indane fragment are rather interesting; for one of those (**37**), detailed pharmacological studies have been carried out [107]. Compound BIIB-104 (PF-04958242, pesampator **38**) is currently in development to target schizophrenia-associated cognitive impairment [3,108]. To date, however, no clinical trials have been conducted with these compounds.

Trifluoromethylpyrazoles are the newest of the classical chemotypes of the AMPA receptor PAMs. Their binding in the central subpocket of the receptor is based primarily on hydrophobic interactions, with the trifluoromethyl group playing a key role (Figure 7B). The main development strategy was a structure-based design starting from compounds found by high-throughput screening. Based on the structural data, modifications were carried out that made it possible to obtain compounds **39–42** with more promising pharmacokinetic characteristics [11,109,110,111,112]. Moreover, a series of hybrid compounds containing an indane sulfonamide fragment (for example, **43** and **44**) and, in some cases, replacing pyrazole moiety with furan, was also developed [11,113].


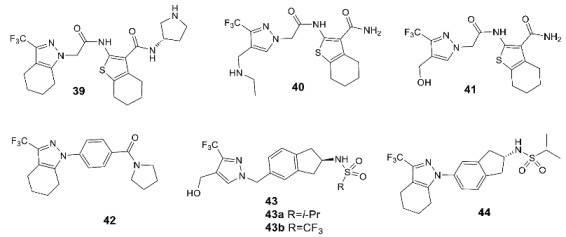


Currently, new chemotypes of the positive allosteric modulators of AMPA receptors are being actively developed. In this respect, great attention is paid to the reduction in side effects, since an expansion of the therapeutic window is required for the clinical use of the compounds. The risk of seizures and other side effects was found to be associated with agonistic effect at high concentrations and a bell-shaped concentration–response relationship [55,68].

Recently, the compound HBT1 (**45**), a trifluoromethylpyrazole modulator, has been found to exhibit lower agonistic effects than other modulators and a normal (sigmoid) rather than a bell-shaped concentration–response relationship [114]. In addition, HBT1 exhibits specific receptor binding, including interaction with the S518 residue. Based on these structural data, promising derivatives of dihydropyridothiadiazine-2,2-dioxide, TAK-137 (**46**) and TAK-653 (**47**), were developed. Their agonistic effect is low, presumably due to the interaction with Ser743 in GluA1 [55,115,116,117].


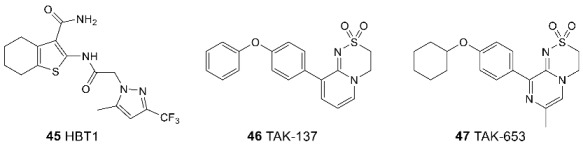


It is important to note that the HBT1 compound in preclinical studies showed pronounced neurotrophic properties and increased BDNF levels in primary neurons [114]. However, at the moment, the exact mechanism of action of these agents remains unknown. 

More extensive preclinical studies have been conducted for TAK-137. It has been shown that this compound can be considered as a drug for the treatment of cognitive impairment in schizophrenia and as a potential new antidepressant [115,118]. In several animal models of schizophrenia, TAK-137 has shown significant improvements in social interaction, working memory, and other cognitive functions [119]. Another recent preclinical study compared the antidepressant properties of TAK-137 and ketamine. It was found that in rats treated with TAK-137 for three days, the same improvements were observed as in the treatment with ketamine, but no psychotomimetic side effects were found [116]. Finally, in preliminary Phase 1 studies with both healthy volunteers and ADHD patients, TAK-137 was found to be safe and well tolerated.

**Figure 8:**
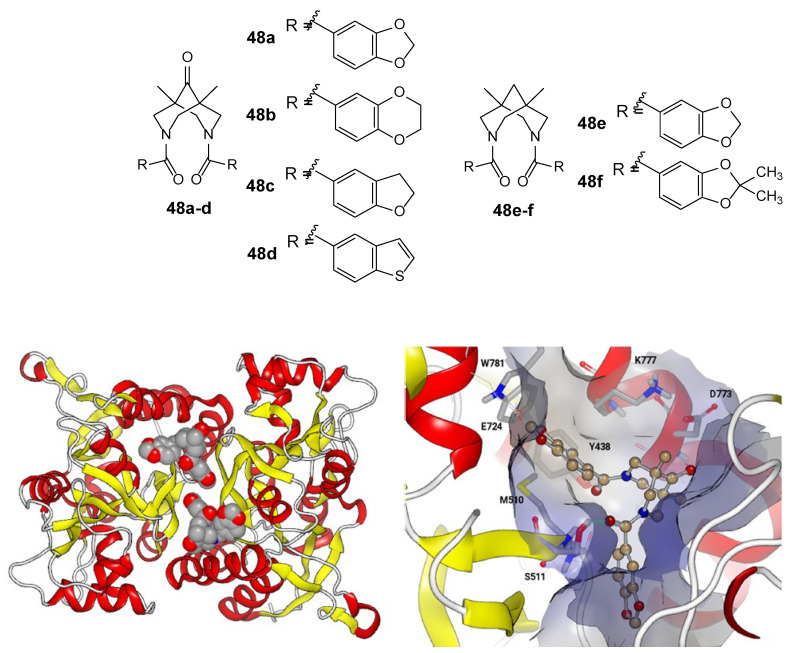
Possible binding mode of compound **48a** in the GluA1 LBD dimer according to molecular docking data [122] (left: symmetrical binding sites of two modulator molecules at the interface of two subunits; right: detailed structure of the binding site). Adapted with permission from Ref. [122]. Copyright 2019, Springer Nature.

A number of interesting structures of AMPA receptor modulators, such as a series of diacyl bispidine derivatives **48,** have been recently developed [120,121,122]. For some of them (**48a**, **48b**, **48c,** and **48e**), the potentiation of the AMPA receptor currents (e.g., for **48a** in the concentration range of 0.01 to 10 nM, with the maximum increase by 110% achieved at 1 nM) and antiamnesic properties in vivo were found [122]; their specific binding to neurons was also studied [123]. According to the updated molecular docking data for these compounds, it is suggested that two modulator molecules bind symmetrically at the interface of the LBD dimer, similar to the binding mode of cyclothiazide (Figure 8) [122].

**Figure 9:**
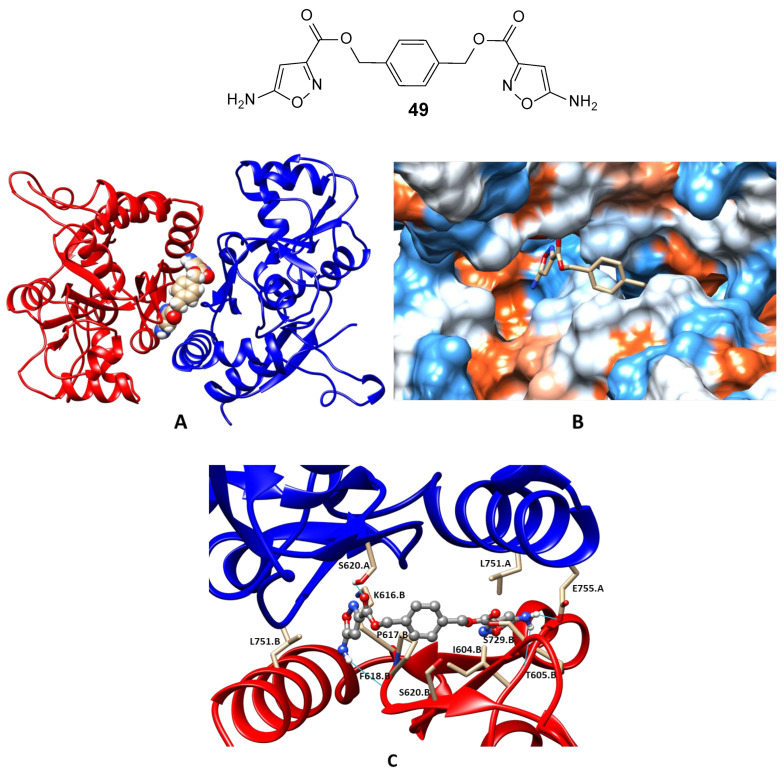
Binding mode of the PAM **49** refined using molecular dynamics simulation. (**A**) General view of the dimeric ligand-binding domain of AMPA receptor (GluA2) and location of the binding site. (**B**) Binding pockets in the protein molecular surface colored by local hydrophobicity (brown for hydrophobic and blue for hydrophilic). (**C**) Detailed view of the binding site. The ligand is represented by a grey ball-and-stick model, the amino acid residues located within 3 Å of it are represented by beige stick models. Hydrogen bonds are shown as cyan lines.

Recently, a new promising series of bis-isoxazoles was obtained and compound **49** demonstrated very high positive modulator activity. The potentiation of the AMPA receptor currents was observed in a wide concentration range (10^−12^−10^−6^ M) and had a bell-shaped concentration dependence with maximum potentiation (up to 72%) at 10^−11^ M [124]. The molecular docking and molecular dynamics studies confirmed that the compound could indeed act as a positive AMPA receptor modulator binding in the validated PAM binding site (Figure 9).

In addition, positive modulatory activity has recently been revealed for a number of well-known compounds that were not previously considered as AMPA receptor ligands. Among the nootropic compounds, we should mention the endogenous peptide cycloprolylglycine **50** and its analogs, for which a potentiating effect on the AMPA receptor (as well as interactions with a number of other targets) has been confirmed [125,126]. Ketamine **51** has been used for several decades as an intravenous anesthetic, and until recently it was considered only as an NMDA receptor blocker. However, in 2000, ketamine was found to exhibit antidepressant activity at subanesthetic concentrations, and in 2019 it was approved by the FDA as a rapid-action antidepressant with several advantages over the previously used drugs. The detailed mechanism of the antidepressant effect of ketamine, its metabolites, and analogs is not fully understood yet, but it has been shown that an important role belongs to the (direct or indirect) potentiation of the AMPA receptors, release or induction of BDNF, and the increase in molecular neuroplasticity [127,128,129,130,131]. Antidepressant effects have also been found for a number of other classes of AMPAR positive allosteric modulators [90,115,132].


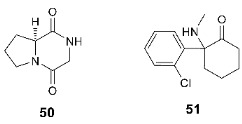


### 3.3. Binding Site for Negative Allosteric Modulators (Non-competitive Antagonists, Non-competitive Inhibitors)

Many of the studied negative allosteric modulators (non-competitive antagonists, non-competitive inhibitors) of the AMPA receptor bind in the linker region between the ligand-binding and transmembrane domains, preventing the displacement of the M3 helix and hindering the ion channel opening. Although for a number of antagonists with a confirmed non-competitive nature of action there is still no information on the binding site or the action mechanism, they will also be considered in this section.

A preeminent example of negative allosteric modulators (noncompetitive antagonists) of the AMPA receptor is perampanel **52** (PMP), approved by the FDA in 2012 as an antiepileptic drug [133]. Since then, research and clinical experience indicate that it is an effective antiepileptic agent with a broad spectrum and a novel mechanism of action [134,135], and may also be useful in alleviating functional and cognitive impairment after stroke [136,137]. In addition, its antitumor activity against brain tumors [138,139] and the possible effectiveness of negative allosteric modulators with a similar mechanism of action against tumors of other organs [140] have been demonstrated.

**Figure 10:**
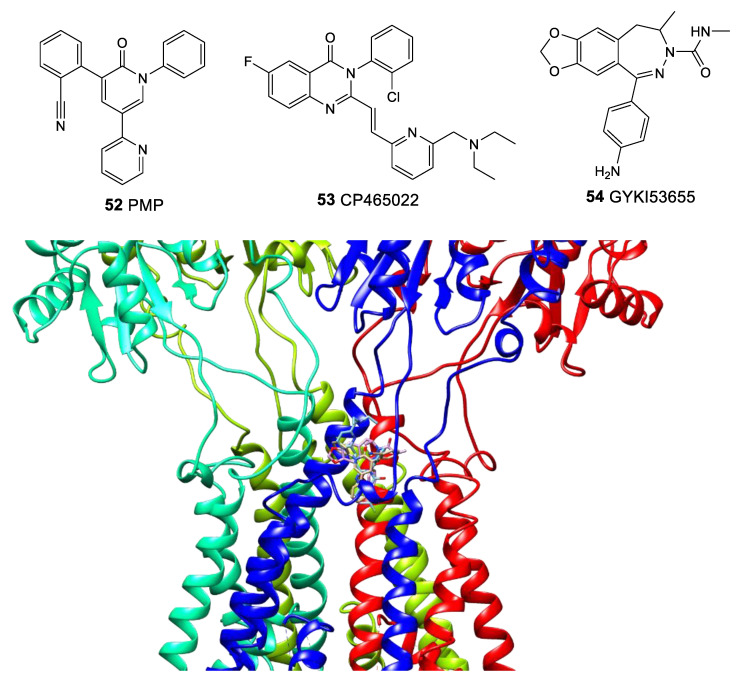
Comparison of the binding modes of negative allosteric modulators GYKI53655 (**54**, pink, PDB: 5L1H), CP465022 (**53**, cyan, PDB: 5L1E), and perampanel (**52**, beige, PDB: 5L1F) at the interface between LBD and TMD of GluA2. For clarity, among the aligned receptor subunits, only the subunits for the perampanel structure are shown in blue, mint, spring green, and red, and only one out of four symmetrical binding positions are shown for each ligand.

Classical chemotypes of negative allosteric modulators of the AMPA receptor are 2,3-benzodiazepines and quinozalin-4-one derivatives. The binding mode and the mechanism of action of such modulators, using CP465022 (**53**), GYKI53655 (**54**), and perampanel as an example, were analyzed in detail by means of the X-ray diffraction analysis, site-directed mutagenesis, molecular and quantum mechanics, molecular docking, and molecular dynamics [141,142,143,144]. All these modulators bind in the interface region of the transmembrane domain (Figure 10). The large size and flexibility of the binding site “pocket”, which is able to adapt to different structures and orientations of the ligands, leads to the binding of modulators with significantly different structures in the same site. Modulator molecules play the role of “wedges” between the transmembrane segments, preventing the M3 helix from shifting when the channel opens. The binding occurs due to multiple weak interactions including hydrophobic contacts, π-stacking, and hydrogen bonds. At the same time, the interaction with certain amino acid residues depends on the state of the receptor, and the significance of some interactions remains debatable.

Despite previous extensive studies of the 2,3-benzodiazepine scaffold, it continues to attract some interest in the search for new negative AMPA receptor modulators. Particularly, in recent years, its isoxazoline derivatives such as **55** [145] and *m*-chlorophenyl analog **56** (the *o*-chloro derivative is inactive) [146] have been obtained.

In addition, new chemotypes of negative allosteric modulators of the AMPA receptor are currently being actively developed. The most interesting among them are the derivatives of phthalazine-1,4-dione (e.g., **57**) [147], pyridothiazinone (e.g., **58**) [148], benzodioxole (e.g., **59**) [149], and 5-chloro-2-oxo-3H-benzoxazole (e.g., **60**) [150]. A non-competitive inhibitory effect and influence on the kinetics of desensitization and deactivation for various subtypes of the AMPA receptor were also found for a number of curcumin derivatives **61** and **62** [151,152,153]. Several promising non-competitive AMPAR antagonists **63** were obtained in the study of arctigenin analogs, with molecular docking results suggesting a binding mode in the transmembrane domain similar to that of the known AMPA receptor non-competitive antagonists such as perampanel [154].


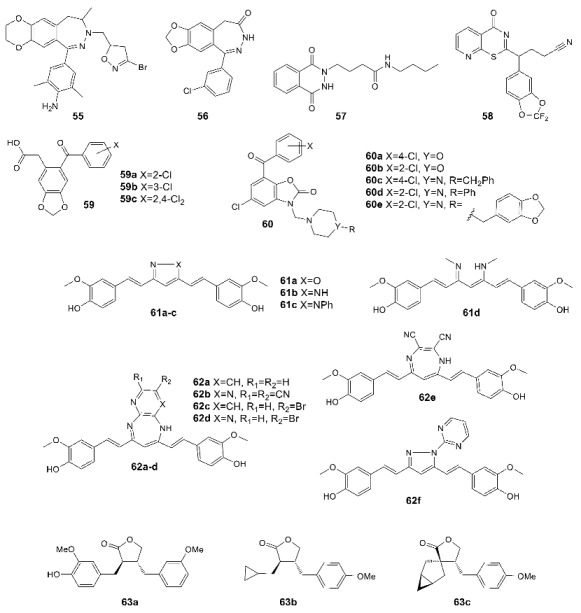


It is interesting to note that the RNA aptamers found by systematic evolutionary selection as well as their chemically modified analogs can also act as noncompetitive inhibitors of AMPA and kainate receptors with different selectivity profiles [155,156,157].

Recently, studies have been published on the simultaneous binding of competitive antagonists and negative allosteric modulators to the AMPA receptor using ZK200775 (**64**) and GYKI53655 (**54**) [50] as an example. It was shown that the interaction of the ligand with the binding site is not affected by the presence of another ligand, and the respective conformational changes occur independently.


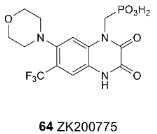


### 3.4. Chemotypes with Activity Cliffs

For some new scaffolds of the allosteric AMPA receptor modulators, the activity cliffs are observed, that is, significant changes in the magnitude and even nature of activity occur with minor changes in the structure of the compound. Possible reasons for this phenomenon are currently under investigation.

For instance, among bis(tetrahydroquinazoline) derivatives [158,159,160], compounds **65a**, **65b**, **65d**, **65e**, **65f**, **65h**, and **65i** are positive modulators of the AMPA receptor in a broad range of concentrations (maximum potentiation by 70%, 55%, 66%, 53%, 77%, 51%, and 61% at 1 nM, respectively), while compounds **65c**, **65g**, and **66** are negative modulators (maximum current reduction by 30% at 0.1 nM; by 50% at 1 μM or 30% at 0.1 nM; EC_50_ = 14 µM, respectively). In the bis(amide) series, compound **67a** is a positive modulator in a broad range of concentrations (maximum potentiation by 40% at 1 nM), while compounds **67b**–**67d** are negative modulators (maximum current decrease at 1 nM by 20%, 50%, and 40%, respectively) [161,162].


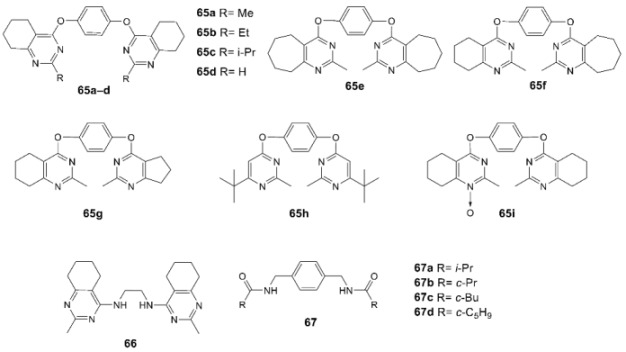


**Figure 11:**
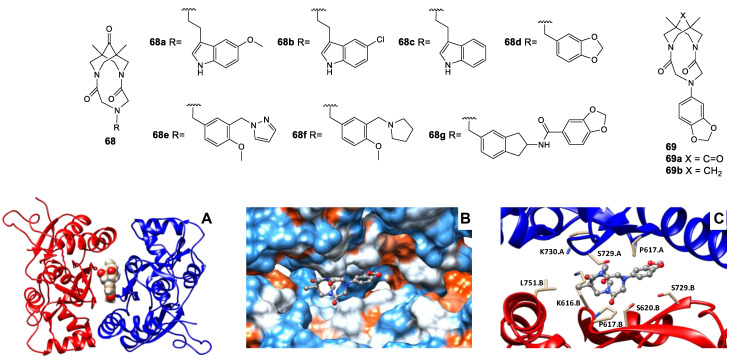
Possible binding mode of the modulator **69a** in the PAM binding site refined using molecular dynamics simulation. (**A**) General view of the dimeric ligand-binding domain of AMPA receptor (GluA2) and location of the binding site. (**B**) Binding pockets in the protein molecular surface colored by local hydrophobicity (brown for hydrophobic and blue for hydrophilic). (**C**) Detailed view of the binding site. The ligand is represented by the gray ball-and-stick model, and the amino acid residues located within 3 Å of it are represented by beige stick models. Adapted with permission from Ref. [167]. Copyright 2022, Elsevier.

Interestingly, in the case of tricyclic scaffold derivatives, an inversion of the modulatory activity can be achieved either by slight structural modifications of the substituent at the N atom or by the presence/absence of a carbonyl group in the bispidine skeleton. Thus, compound **68a** is inactive, compounds **68b**, **68e**, and **68g** are positive modulators of the AMPA receptor in a wide concentration range (maximum potentiation by 30% at 1 nM, 40% at 0.001 nM, and 65% at 1 nM, respectively), and compounds **68c**, **68d**, and **68f** are negative modulators (maximum current decrease by 20% at 0.1 nM, 35% at 0.01 nM, and 35% at 1 nM, respectively) [163,164,165,166]. To date, compounds **68e** and **68f** have undergone a full cycle of preclinical trials, the results of which are being prepared for publication. Compound **69a** is a highly potent positive AMPA receptor modulator (maximum potentiation of 62% at 1 nM), while compound **69b** is a negative modulator (maximum current reduction of 39% at 0.1 μM) [167]. Based on the molecular docking and molecular dynamics results, compound **69a** can indeed interact with the validated PAM binding site at the interface between the dimeric LBDs (Figure 11) while the binding stability of compound **69b** is much lower and it should have substantially weaker PAM activity. On the other hand, it is suggested that compound **69b** can be tentatively expected to act via the NAM binding site at the LBD–TMD interface.

### 3.5. Binding Site for TARP-Dependent Allosteric Modulators

Currently, the study of the binding sites for TARP-dependent allosteric modulators remains a serious problem. Particularly, the difficulties are caused by the variability of the complexes of auxiliary TARP proteins with AMPA receptor subunits (Figure 3) [168,169,170,171] and, sometimes, by an incomplete understanding of the physiological relevance of potential variations, as well as the occurrence of a variety of physiological effects. It was also found that the interaction of TARP subunits with AMPA receptors substantially affects the conductance of the ion channel [171].

Nevertheless, in recent years, the application of advanced experimental and molecular modeling techniques has enabled significant progress in the determination of the structural features of TARP subunits and the mechanisms of their action on the AMPA receptors [168,172,173,174,175]. For example, according to molecular modeling data, known TARP γ8-dependent negative allosteric modulators of the AMPA receptor bind at the interface between interacting transmembrane segments of TARP and the receptor [176]. One of the modulator molecule fragments immobilizes it by “wedging” into the pocket between the TM3 and TM4 transmembrane segments in the TARP structure, which expands during thermal motion, while the rest of the molecule is exposed to the area of contact with the M1 peripheral transmembrane segment of the receptor, leading to disruption of their interaction and blocking the positive modulating effect of TARP. This results in decreasing the probability of channel opening to high-conductivity states and reduces the weighted mean single-channel conductance without affecting recovery from desensitization [177].

As already noted, due to the differences in the expression of TARP proteins in different functional parts of the brain, modulators that are selective for certain subtypes provide the possibility of targeted control of the pharmacological effect [33,34]. In this regard, the problem of searching for selective TARP-dependent modulators has attracted considerable interest in recent years. To solve it, as a rule, high-throughput screening methods are used, followed by optimization of activity and pharmacokinetic properties. Most of the currently known ligands are TARP γ8-dependent negative allosteric modulators of the AMPA receptor **70–73** [178,179,180,181], which can potentially be used to create antiepileptic drugs with minimal side effects. Despite noticeable structural differences, they all contain an oxindole fragment (or its hetero analog), which plays the role of a fixing “wedge” during ligand binding (see above). 


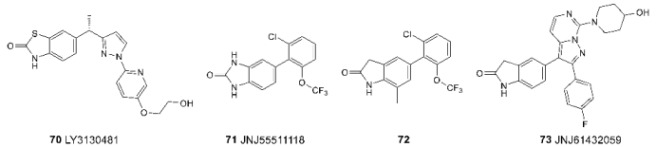


With the aid of a specially developed high-throughput screening procedure using voltage-sensitive dyes and fluorescent detection of calcium ion penetration into the cell, modulators selective to other auxiliary proteins of AMPA receptors were also found [182]. Compounds **74** and **75** act as negative modulators, while compound **76** is a positive modulator for the receptor complexes with stargazine (TARP γ2) and CNIH3. 


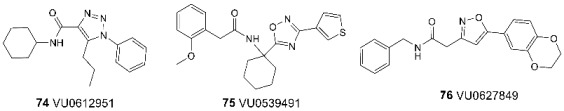


It should be noted that this area is a focus of expanding ongoing research that aims to study the mechanisms of interactions between AMPA receptor ligands, individual receptor subunits, and various auxiliary proteins leading to specific therapeutic effects.

### 3.6. Binding Sites for Ion Channel Blockers 

Blocking of the ion channel pore by positively charged small molecule ions is apparently important only for the AMPA receptor forms that have no region of increased positive charge associated with arginine residues in their pores and are therefore permeable to calcium ions [19,183]. Endogenous polyamines spermine **77a**, spermidine **77b**, and putrescine **77c**, protonated at physiological pH, can act as such blockers, predominantly localized inside the cell. The degree of blocking depends on the membrane potential and is usually negligible under normal physiological conditions; however, they provide additional possibilities for controlling the permeability of the channel. Interestingly, in the case of NMDA receptors, polyamines also act as GluN2B-selective positive modulators interacting with the amino-terminal domain [184].


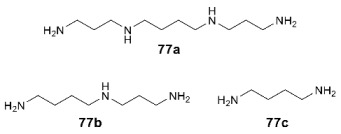


**Figure 12:**
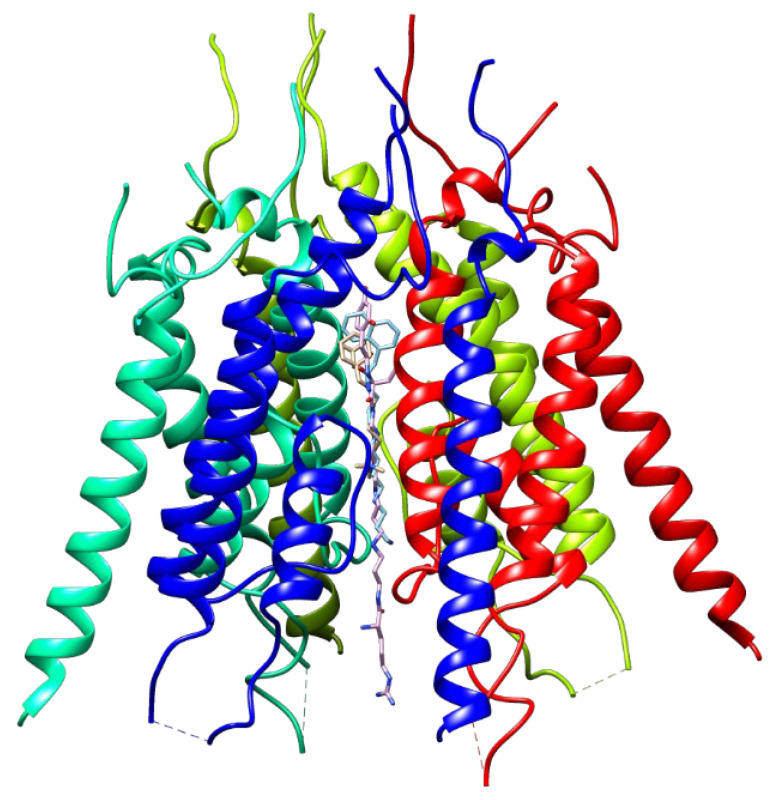
Binding modes of the AMPA receptor ion channel blockers IEM-1460 (**83**, beige, PDB: 6DM0), NASPM (**80**, cyan, PDB: 6DM1), and AgTx-636 (**78**, pink, PDB: 6O9G) in the ion channel pore of GluA2 TMD. For clarity, among the aligned receptor subunits, only the subunits for the IEM-1460 structure are shown in blue, mint, spring green, and red.

Under physiological conditions, at a negative membrane potential, Ca^2+^-permeable AMPA receptors can be blocked by the extracellular exogenous molecules that also usually contain a polyamine or acylpolyamine chain, often linked to a bulky hydrophobic group. Such blockers can be natural toxins (e.g., argiotoxin-636 **78** from the venom of *Argiope lobata* spiders [19] or philanthotoxin-433 **79** from the venom of *Philanthus triangulum* wasp [185]), their semi-synthetic or synthetic analogs (e.g., naphthylacetylspermine **80** and philanthotoxin analogs **81**, **82** [185,186]), or similarly constructed fully synthetic structures (e.g., adamantane derivative **83**). Like other AMPA and NMDA receptor antagonists and blockers, they could potentially have antiepileptic and neuroprotective properties. When a blocker molecule binds, its hydrophobic group is located in the central cavity of the ion channel pore (above the selectivity filter formed by the M2 segments) while the polyamine chain passes through the negatively charged filter pore toward the inside of the cell (Figure 12) [19,183].

By modifying the structure of argiotoxin, a reversible photoswitchable blocker **84** of the Ca^2+^-permeable AMPA receptor channel was developed [187]. In the dark, it has an active *trans*-form, and when irradiated with a 470 nm wavelength light, it passes into an inactive *cis*-form, which allows precise and non-invasive control of the receptor operation in space and time.

A novel chemotype of non-competitive AMPA receptor antagonists was recently found in the study of diminazene **85**, a diarylamidine anti-infective agent [188]. Electrophysiological studies indicate that its mechanism of action likely involves blocking the AMPAR ion channels, perhaps accompanied by penetration through the channel pore into the cell. In addition, diminazene is known to block the NMDA receptor and the acid-sensing ion channels (ASICs) while the elongated general shape of the molecule and its positive charge at physiological pH are similar to the classical AMPA receptor channel blockers.


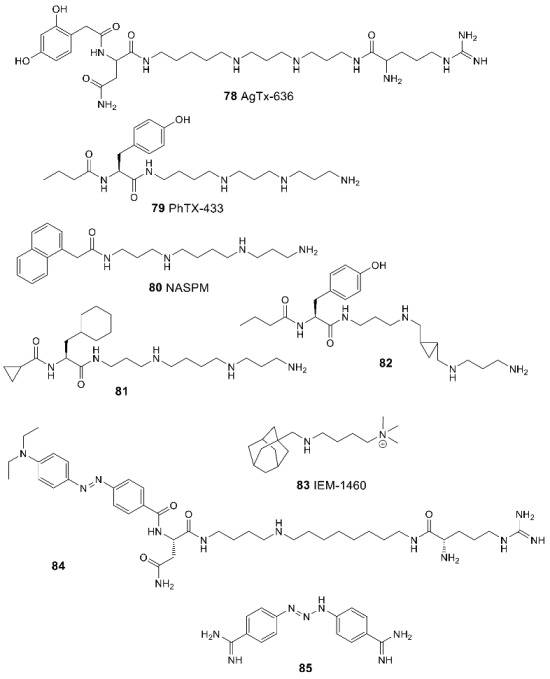


## 4. Conclusions

AMPA receptors, like other ionotropic glutamate receptors, have attracted considerable attention for several decades and remain the subject of intense research. They play a key role in providing synaptic plasticity, which is one of the mechanisms of learning and memory formation, and can also act as targets for the creation of new drug classes for the treatment or significant correction of a number of serious neurodegenerative and neuropsychiatric diseases. At the same time, this potential and the enormous effort expended by many researchers around the world have so far been embodied in specific drugs used in clinical practice only to a very limited extent. In part, this may be due to the complexity of the human nervous system and, in particular, the glutamatergic system, as many aspects of its operation are revealed only gradually. Nevertheless, the vast factual and theoretical material accumulated in recent years, as well as the successes of structural biology, neurobiology, molecular modeling, and medicinal chemistry, allow us to expect significant progress in this area in the near future.

## Data Availability

Not applicable.
